# Adaptation to mis-pronounced speech: evidence for a prefrontal-cortex repair mechanism

**DOI:** 10.1038/s41598-020-79640-0

**Published:** 2021-01-08

**Authors:** Esti Blanco-Elorrieta, Laura Gwilliams, Alec Marantz, Liina Pylkkänen

**Affiliations:** 1 Department of Psychology, New York University, New York, NY 10003 USA; 2 Department of Linguistics, New York University, New York, NY 10003 USA; 3 NYUAD Institute, New York University Abu Dhabi, P.O. Box 129188, Abu Dhabi, UAE; 4 Department of Psychology, Harvard University, Cambridge, MA 02138 USA; 5 Department of Neurological Surgery, University of California San Francisco, San Francisco, CA 94115 USA

**Keywords:** Language, Perception

## Abstract

Speech is a complex and ambiguous acoustic signal that varies significantly within and across speakers. Despite the processing challenge that such variability poses, humans adapt to systematic variations in pronunciation rapidly. The goal of this study is to uncover the neurobiological bases of the attunement process that enables such fluent comprehension. Twenty-four native English participants listened to words spoken by a “canonical” American speaker and two non-canonical speakers, and performed a word-picture matching task, while magnetoencephalography was recorded. Non-canonical speech was created by including systematic phonological substitutions within the word (e.g. [s] → [sh]). Activity in the auditory cortex (superior temporal gyrus) was greater in response to substituted phonemes, and, critically, this was not attenuated by exposure. By contrast, prefrontal regions showed an interaction between the presence of a substitution and the amount of exposure: activity decreased for canonical speech over time, whereas responses to non-canonical speech remained consistently elevated. Grainger causality analyses further revealed that prefrontal responses serve to modulate activity in auditory regions, suggesting the recruitment of top-down processing to decode non-canonical pronunciations. In sum, our results suggest that the behavioural deficit in processing mispronounced phonemes may be due to a disruption to the typical exchange of information between the prefrontal and auditory cortices as observed for canonical speech.

## Introduction

Speech provides the ability to express thoughts and ideas through the articulation of sounds. Despite the fact that speech sounds are often ambiguous, humans are able to decode these signals and communicate efficiently.

This ambiguity emerges from a complex combination of physiological and cultural features^1–6^; and results in situations where, for example, one talker’s “ship” is physically very similar to another speaker’s “sip”^7^. Indeed, any given sound has a potentially infinite number of acoustic realizations^8,9^; comprehending speech therefore necessitates accommodating for the discrepancy between the perceived phoneme and the personal ideal form of that phoneme. This process is known as perceptual attunement^10^.

One prevalent example of such need for accommodation occurs when listening to an accented talker^11^, and involves adjusting the mapping from acoustic input to phonemic categories through perceptual learning^10,12–16^; see^17,18^ for a review). This adjustment can happen quickly: the lower bound is estimated to be approximately 10 sentences for non-native speech^19,20^, and within 30 sentences for noise-vocoded speech^21^.

While adaptation to non-canonical speech has been robustly reported behaviorally, to date, the majority of work examining the neural underpinnings of this process has focused on distorted or degraded speech, rather than systematic phonetic variation (e.g.^21–23^). Importantly, these two types of manipulations tap into fundamentally different phenomena: a signal-to-noise problem in the degraded case, and a mapping problem in the variation case. So far, it is known that the patterns of activation underlying the perception of speech in noise and accented speech dissociate^24^, and that they vary when listening to regional as compared to foreign accents^25^. Accented speech appears to recruit anterior and posterior superior temporal gyrus (STG), planum temporale (PT) and frontal areas including inferior frontal gyrus (IFG)^24^. However, little is known about how responses in these areas are modulated during exposure to systematic phonological variation—perhaps neural processing differences disappear after a sufficient listening period, for example.

Much more is known about how *canonically* pronounced phonemes are neurally processed. Early responses around 100–200 ms in STG have been linked to a bottom-up “acoustic–phonetic mapping”^26,27^. At this latency, neural responses respond to the phonetic categories^28^ and are not influenced by higher-order information such as word identity. Neural responses later than 200 ms are instead associated with higher-order processing, such as the integration of previous expectations in STG (e.g., phoneme surprisal;^29–31^) and integration of lexical information in middle temporal gyrus^32,33^.

The goal of our study is to understand what processing stage is involved in adaptation to systematically mispronounced speech. If we observe that STG responses at 100–200 ms vary as a function of exposure, we may conclude that adaptation serves to directly alter the mapping between the acoustic signal and its phonetic features. This would support a “neural recalibration hypothesis”. If, alternatively, exposure only modulates later neural responses, potentially in higher-order cortical areas, the process may be better accounted for by a “repair hypothesis” that updates the phonetic features that were incorrectly identified during the initial bottom-up computation.

To this end, 24 native English participants listened to isolated words that were either pronounced canonically (baseline condition), or were pronounced with systematic phonological substitutions. We consistently assigned different speakers to canonical and non-canonical pronunciations, such that listeners would learn the mapping between speaker identity and expected pronunciation. Each experimental block contained 20 words all produced by the same speaker, allowing speaker identity to influence the processing of all subsequent critical phonemes. Neural responses were recorded using magneto-encephalography (MEG), while participants performed a word-picture matching task, and were analyzed at the single-trial level.

## Methods

### Participants

We tested 24 subjects (11 male, 13 female; *M* = 27.8 years, *SD* = 9). All participants were right-handed, monolingual native English speakers with no history of neurological anomalies and with normal or corrected-to-normal vision. All participants received monetary compensation or course credit for their participation and provided informed written consent following NYU Institutional Review Board protocols. This study was performed according to the NYU Institutional Review Board protocols and to the Declaration of Helsinki.

### Stimuli

**Figure 1:**
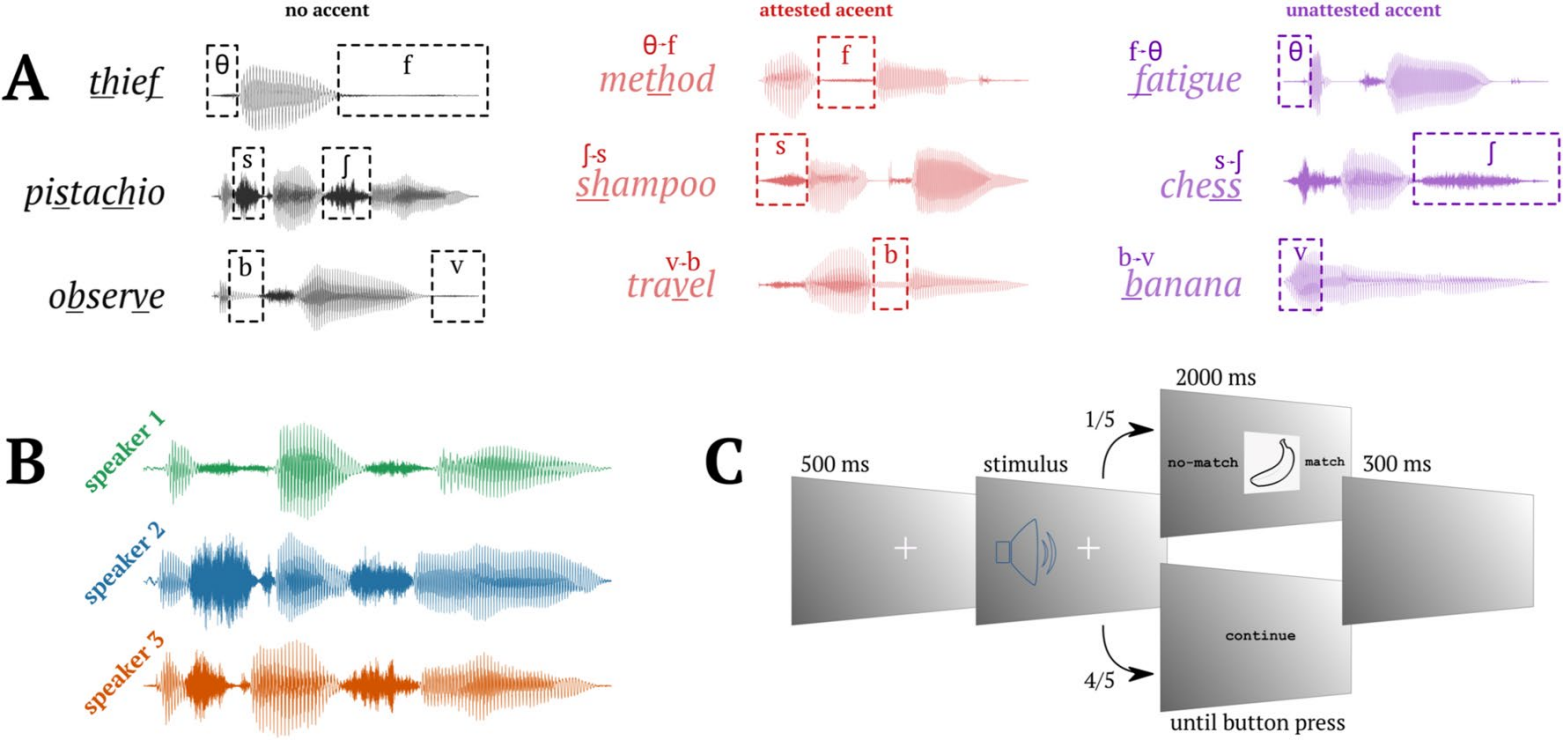
Experimental stimuli and design. (**A**) Experimental conditions. In black, left column: baseline condition. In red, middle column: attested substitutions. In purple, right column: unattested substitutions. (**B**) Waveform of three speakers’ pronunciation of *pistachio*. (**C**) Trial structure.

In order for the experiment to be informative of non-canonical speech processing, we selected three phonemic minimal-pairs that are often transposed in certain variations of English (Attested variation: v → b substitutions (attested in Spanish speakers of English); θ → f substitutions (th-fronting, attested in English speakers from London) and ʃ → s substitutions (attested in Indian speakers of English; Fig. 1A). Importantly, these phoneme substitutions are systematic for speakers of those variations and occur uni-directionally (e.g., native Spanish speakers of English often substitute instances of /v/ with /b/, but not vice versa). We created 240 words containing these “Attested” substitutions.

We also presented participants with substitutions in the reverse direction, which are not naturally occurring and thus, participants could not have previously ascribed to specific dialectal or sociolectal variations (Unattested variation; b → v, f → θ and s → ʃ). We created 240 words containing these “Unattested” substitutions. Using the same phonemes in both conditions allowed us to keep the perceptual distance in the two conditions constant, ensuring that any observed differences between the two conditions were due to the level of initial attunement and not this confounding factor.

Lastly, our experiment included a baseline condition where all phonemes were pronounced accurately and no adaptation was required (Baseline condition; for examples of stimuli for each condition please see Fig. 1A). We selected 120 “Baseline” words, each containing the correct pronunciation of two of the six phonemes manipulated in the Attested and Unattested conditions (i.e. any two of: b, v, f, θ, s, ʃ).

In total, there were 720 critical phonemes presented within 600 words. Critical phonemes were presented embedded in meaningful words, and all words were selected such that the critical phoneme substitution created non-words (e.g., the word *travel* becomes a non-word if critical phoneme /v/ is substituted by /b/ to form *trabel*). There were two reasons for selecting these types of words: (1) to avoid cases where not adapting to the phoneme substitutions would still result in a meaningful word (and thus the substitution would go unnoticed) and (2) to take advantage of the Ganong effect (Ganong, 1980), which establishes that listeners tend to make phonetic categorizations that make a word of the heard strings. It follows then that using cases where only one of the interpretations results in a meaningful word should maximize the chances of listeners’ adaptation.

Importantly, words were selected such that the six critical phonemes only occurred in the conditions of interest (e.g., if /s/ was a phoneme we were interested in, this phoneme only occurred in the condition of interest and not in the others). This selection process exhausted the pool of English words that met all of our stimuli selection criteria. For a complete list of experimental items please see Additional Materials 1.

All stimulus words had a minimum log surface frequency of 2.5 in the English Lexicon Project corpus^34^, were between 3 and 10 phonemes long, and were monomorphemic. Words across conditions were matched for log surface frequency (M = 8.19, SD = 1.92), length (M = 4.51, SD = 1.36) and critical phoneme position (M = 1.64, SD = 1.36). All critical phonemes appeared the same number of times in the experiment.

### Experimental design

Three native speakers of American English were recorded to create the stimuli, all of them female but with distinctive voices (F0 speaker 1 M = 205 Hz, SD = 13 Hz; speaker 2 M = 200 Hz, SD = 21 Hz; speaker 3 M = 184 Hz, SD = 31 Hz). Each individual was recorded saying all 600 items, following the variation rules previously explained for the items in the Attested and Unattested conditions, and naturally pronouncing the Baseline words (Fig. 1B). Stimuli were recorded in a single recording session for each speaker. Each word was read three times, and the second production of the word was always selected to allow for consistent intonation across stimuli. Critical phoneme onsets were identified manually and marked using Praat software^35^.

The assignment of speaker to condition was assigned across participants following a latin-square design. This means that each participant only heard each type of systematic mispronunciation (or, lack thereof) by one speaker (e.g. for participant A: attested = speaker1; unattested = speaker2; baseline = speaker3; for participant B: attested = speaker2; unattested = speaker3; baseline = speaker1, etc.). Items for each condition were further divided into blocks of 20 items to form experimental blocks. Items within blocks were fully randomized, and blocks within the experiment were pseudo randomized following one constraint: Two blocks of the same condition never appeared successively. Each stimulus was presented once during the experiment and each participant was presented with a unique order of items. Stimuli were presented binaurally to participants through tube earphones (Aero Technologies), using Presentation stimulus delivery software (Neurobehavioral Systems).

### Experimental procedure

**Figure 2:**
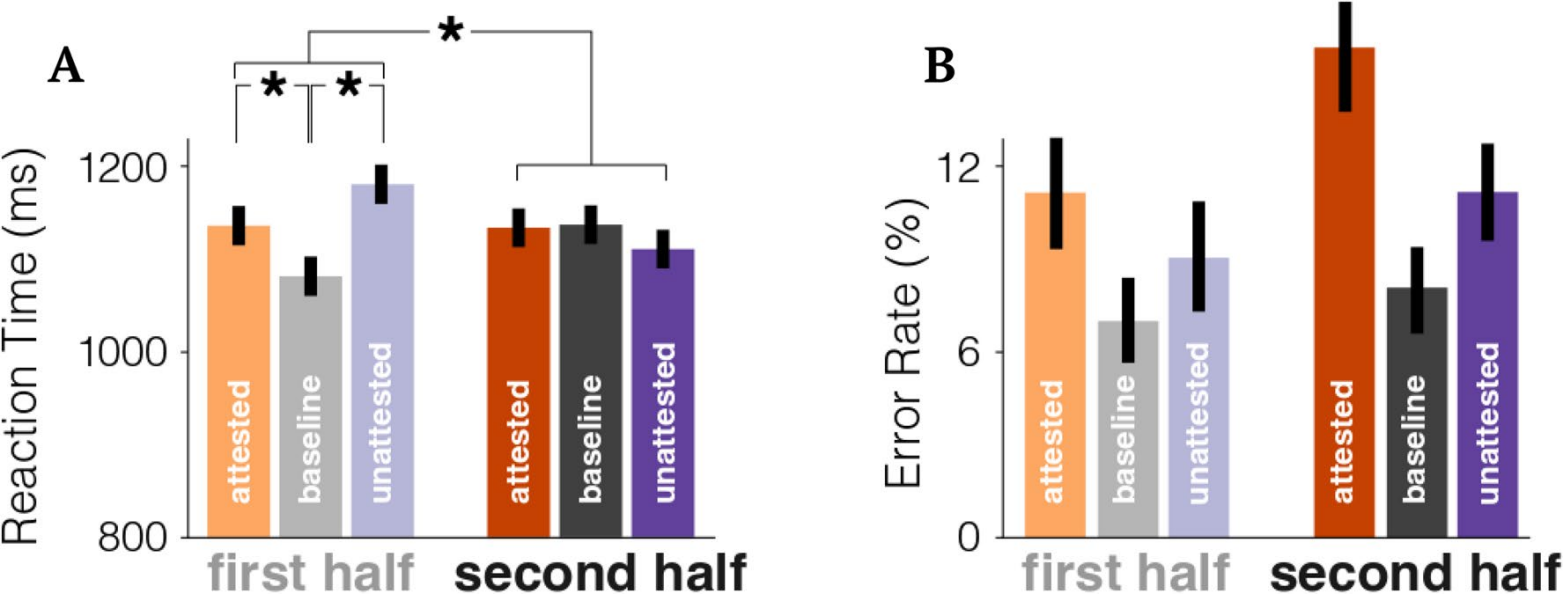
Behavioral responses to the word-picture matching task. (**A**) Behavioral reaction times showing that people are slower identifying pictures at the beginning of the experiment when the word was pronounced non-canonically, but adjust to it as the experiment goes on. The results show a main effect of condition, and an interaction such that attested and unattested conditions are responded to significantly faster as a function of exposure. (**B**) Error rates showing that participants made numerically more mistakes for non-canonical speech, though this difference is not significant—regardless of exposure. Note that the statistical analysis was based on a continuous measure of exposure, but trials are grouped here for illustrative purposes.

Each trial began with the visual presentation of a white fixation cross on a gray background (500 ms), which was followed by the presentation of the auditory stimulus. In one fifth of the trials, 500 ms after the offset of the auditory stimulus, a picture appeared on the screen (see Fig. 1C). Participants were given 2000 ms to make a judgment via button press to indicate whether the picture matched the word they had just heard. For all participants, the left button indicated “no-match” and the right button indicated “match”. In the other four fifths of the trials, participants would see the word “continue” instead of a picture, which remained on-screen until they pressed either button to continue to the next trial. The picture-matching task was selected to keep participants engaged and accessing the lexical meaning of the stimuli while not explicitly drawing attention to the phoneme substitutions. The 500 ms lapse between word offset and the visual stimulus presentation was included to ensure that activity in our time-windows of interest was not interrupted by a visual response. In all trials, the button press was followed by 300 ms of blank screen before the next trial began (Fig. 2B). Behavioral reaction times were measured from the presentation of the picture matching task. The study design and all the experimental protocols carried out during the study were approved by the NYU IRB committee.

### Data acquisition and preprocessing

Before recording, each subject’s head shape was digitized using a Polhemus dual source handheld FastSCAN laser scanner (Polhemus, VT, USA). Digital fiducial points were recorded at five points on the individual’s head: The nasion, anterior of the left and right auditory canal, and three points on the forehead. Marker coils were placed at the same five positions in order to localize that person’s skull relative to the MEG sensors. The measurements of these marker coils were recorded both immediately prior and immediately after the experiment in order to correct for movement during the recording. MEG data were collected in the Neuroscience of Language Lab in NYU Abu Dhabi using a whole-head 208 channel axial gradiometer system (Kanazawa Institute of Technology, Kanazawa, Japan) as subjects lay in a dimly lit, magnetically shielded room.

MEG data were recorded at 1000 Hz (200 Hz low-pass filter), and noise reduced by exploiting eight magnetometer reference channels located away from the participants’ heads via the Continuously Adjusted Least-Squares Method^36^, in the MEG Laboratory software (Yokogawa Electric Corporation and Eagle Technology Corporation, Tokyo, Japan). The noise-reduced MEG recording, the digitized head-shape and the sensor locations were then imported into MNE-Python^37^. Data were epoched from 200 ms before to 600 ms after critical phoneme onset. Individual epochs were automatically rejected if any sensor value after noise reduction exceeded 2500 fT/cm at any time. Additionally, trials corresponding to behavioral errors were also excluded from further analyses.

Neuromagnetic data were coregistered with the FreeSurfer average brain (CorTechs Labs Inc., La Jolla, CA and MGH/HMS/MIT Athinoula A. Martinos Center for Biomedical Imaging, Charleston, MA), by scaling the size of the average brain to fit the participant’s head-shape, aligning the fiducial points, and conducting final manual adjustments to minimize the difference between the headshape and the FreeSurfer average skull. Next, an ico-4 source space was created, consisting of 2562 potential electrical sources per hemisphere. At each source, activity was computed for the forward solution with the Boundary Element Model (BEM) method, which provides an estimate of each MEG sensor’s magnetic field in response to a current dipole at that source. Epochs were baseline corrected with the pre-target interval [− 200 ms, 0 ms] and low pass filtered at 40 Hz. The inverse solution was computed from the forward solution and the grand average activity across all trials, which determines the most likely distribution of neural activity. The resulting minimum norm estimates of neural activity^38^ were transformed into normalized estimates of noise at each spatial location, obtaining statistical parametric maps (SPMs), which provide information about the statistical reliability of the estimated signal at each location in the map with millisecond accuracy. Then, those SPMs were converted to dynamic maps (dSPM). In order to quantify the spatial resolution of these maps, the pointspread function for different locations on the cortical surface was computed, which reflects the spatial blurring of the true activity patterns in the spatiotemporal maps, thus obtaining estimates of brain electrical activity with the best possible spatial and temporal accuracy^39^. The inverse solution was applied to each trial at every source, for each millisecond defined in the epoch, employing a fixed orientation of the dipole current that estimates the source normal to the cortical surface and retains dipole orientation.

### Statistical analysis

#### Behavioral analysis

First, we tested for a behavioural correlate of speaker adaptation: in this case, faster responses and fewer error rates in the picture-matching task as a function of exposure to non-canonical pronunciations. We performed a basic cleaning on the data, removing trials that were responded to incorrectly or faster than 300 ms. Then, we fit a linear mixed effects regression model, including fixed effects for condition (with three levels: baseline, attested, unattested); numerical count of exposures to the speaker; number of elapsed trials; whether they were indicating a match or mis-match with the picture, and speaker identity. Critically, we also included the interaction between condition and number of exposures—this is the effect we would expect to see if subjects are indeed attuning to the mispronounced speech. The model also included a random slope for trial and subject, and a full random effects structure over items for all of the fixed effects.

#### Regression on source localised MEG data: ROI analysis

We ran a similar linear mixed effects regression model as the one described above, time-locked to the onset of each critical phoneme (i.e., fixed effects for condition; numerical count of exposures to a certain speaker; number of elapsed trials across all three speakers; picture match or mis-match, and speaker identity). In addition, we also added phoneme position in the word as a covariate in order to account for global magnitude differences that arise from distances from word onset. Based on previous research^26,27^, the model was fit on average responses in transverse temporal gyrus and superior temporal gyrus in the left and right hemispheres separately (our lower-level regions of interest (ROIs)). And then separately in the orbito-frontal cortex, inferior frontal gyrus (IFG), dorsolateral prefrontal cortex (dlPFC) bilaterally (our higher-order ROIs;^40^), We used *freesurfer* to parcellate the average brain (using the *aparc* parcellation, based on the Desikan-Killiany Atlas) and to index the vertices corresponding to each region. Activity was averaged within each ROI, and the model was fit at each millisecond, to yield a time-series of model coefficients for each variable of interest, using the *lme4* package in *R*^41^. The model included a random slope for trial and subject, and a full random effects structure over items for all of the fixed effects.

These model coefficients were then submitted to a temporal cluster test in order to estimate during which time window(s) a variable significantly modulated neural responses. Concretely, this involves testing, at each time sample, whether the distribution of coefficients over subjects is significantly different from chance using a one-sample t-test to yield a timecourse of t-values. Clusters are formed based on temporally adjacent t-values that exceed a *p* < 0.05 threshold. We sum the t-values within a cluster to form the test statistic. Next, to form a null distribution, we randomly permute the sign of the beta coefficients within the cluster extent and re-compute the test statistic (the largest observed summed t-value in a cluster) 10,000 times. The proportion of times that the observed test statistic exceeded samples of the null distribution forms our *p* value. This procedure corrects for multiple comparisons across time samples (family wise error *p* < 0.05). For this, we used the *mne-python* module^37^, which follows the approach of non-parametric analyses outlined in^42^.

#### Connectivity analysis

We used Wiener–Granger causality (G-causality;^43,44^) to identify causal connectivity between our different regions of interest in the MEG time series data. This analysis was conducted using the Multivariate Granger Causality Matlab Toolbox^45^. The input to this analysis was the time course of activity averaged over all the sources in each Brodmann area of interest. Based on these time series data, we first calculated the Akaike information criteria (AIC;^46^) and Bayesian information criteria (BIC;^47^) from our time series data using Morf’s version of the locally weighted regression^48^. Then, we fitted a VAR model to our time series data, using the best model (AIC or BIC) order as determined in the previous step, applying an ordinary least squares regression. At this point we checked for possible problems in our data (non-stationarity, collinearity, etc.), and after ensuring that our time series fulfilled all statistical assumptions, we continued to calculate the auto-covariance sequence, using the maximum number of auto-covariance lags. Finally, from the sequence of auto-covariance we calculated the time-domain pairwise-conditional Granger causalities in our data. Pairwise significance was corrected using FDR^49^ at an alpha value of *p* = 0.01.

## Results

### Behaviour

Our regression on reaction times revealed no main effect of condition (χ^2^ = 3.23, *p* = 0.19), number of exposures (χ^2^ = 0.46, *p* = 0.49) or speaker identity (χ^2^ = 0.95, *p* = 0.62). There were main effects of number of elapsed trials in total (collapsed across speakers) (χ^2^ = 3.9, *p* = 0.048) and match/mismatch response (χ^2^ = 35.34, *p* < 0.001). Critically for our analysis, there was a significant interaction between condition and number of exposures (χ^2^ = 9.96, *p* < 0.01). This interaction is driven by the speed-up in responses to attested and unattested substitutions as a function of exposure: There was a significant effect of number of exposures when just analyzing the attested and unattested trials (χ^2^ = 5.5, *p* = 0.019), but no significant effect of exposure for just the baseline speech (χ^2^ = 1.9, *p* = 0.17). Average behavioural responses are shown in Fig. 2A, split between first and last half of the experiment for illustrative purposes.

We then fit the same model to the accuracy data. The only significant factor was match/mismatch response, whereby participants were significantly less accurate in mis-match trials (χ^2^ = 147.19, *p* < 0.001). Although participants were on average more accurate in baseline trials than in non-canonical trials baseline = 92.9%, attested = 87.1%, unattested = 90.4% the model did not reveal a significant effect of condition (χ^2^ = 2.51, *p* = 0.28) nor an interaction with the amount of exposure (χ^2^ = 1.28, *p* = 0.53). There was also no main effect of number of exposures (χ^2^ = 0.0026, *p* = 0.96).

### Neural results

Identifying a behavioral effect of adaptation serves as a necessary sanity check that our experimental paradigm is fostering adaptive learning in our participants; however, it is not sufficient to inform *how* adaptation happens. For this, we tested for neural responses that mirror the observed behavioral adaptation, relative to our two hypotheses. If we find that 100–200 ms responses in auditory cortex are modulated by exposure, this would support the recalibration hypothesis. By contrast, if we find that later responses in higher-level areas (i.e. in frontal regions) are modulated by exposure, this would support the repair hypothesis.

We adjudicated between these two possibilities by fitting the same linear regression model used to analyze our behavioral data to the source-localized neural activity.

**Figure 3:**
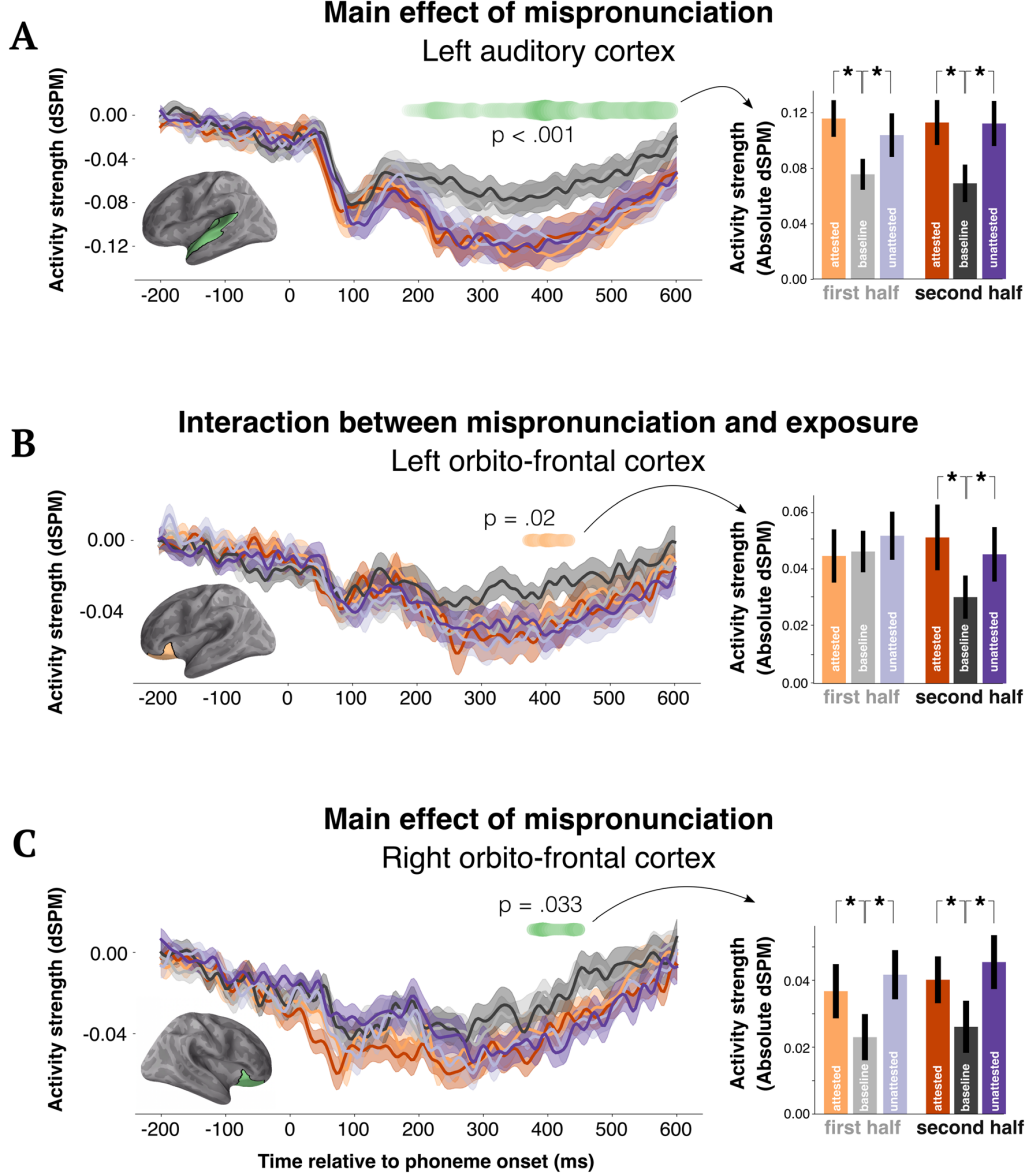
Average condition averages in regions of interest, with regression coefficients over time for a multiple regression model with factors: numerical count of exposures to the speaker; number of elapsed trials; picture match or mismatch, speaker identity and condition (baseline, attested, unattested). In each panel, the brain model shows the location of the area where the analysis was conducted. Barplots show absolute activity averaged over the temporal window of significance and over sources in the tested ROI. Note that the activity in the time-courses is “signed”, so has both positive and negative fluctuations, whereas in the right barplots we just show the absolute values of the activity so that the effects are directly visually comparable to the behavioral figures. Panel (**A**) shows a main effect of mispronunciation: regardless of exposure, non-canonical speech always elicits more activity in auditory cortices. Panel (**B**) shows an interaction effect whereby prefrontal signals decrease for baseline phonemes over exposure whereas non-canonical speech requires its engagement. Panel (**C**) shows a main effect of mispronunciation in the right orbital-frontal cortex, similar to the pattern we observe in auditory cortices.

In the left auditory cortex, we found a main effect of condition, such that attested and unattested substitutions consistently elicited more activity as compared to canonical pronunciations, between 188–600 ms (summed uncorrected t-values over time = 51.57; *p* = 0.002; Fig. 3B). There was no interaction with exposure (sum t-value = 6.59), and indeed, numerically the magnitude of the effect remained stable from the first to the second half of the experiment. There were no effects in right auditory cortex.

Within the areas analyzed in the left frontal cortex, there was no main effect of exposure (no temporal clusters formed), but there was a significant interaction between condition (baseline, attested or unattested) and exposure in the left orbitofrontal cortex from 372 to 436 ms after phoneme onset (sum t-value = 8.65; *p* = 0.02; Fig. 3A). The pattern of the response was such that in the first half of the experiment, all conditions elicited a similar response magnitude, but over exposure, responses to the canonically pronounced phonemes significantly decreased as compared to substituted phonemes. In the right orbitofrontal cortex, we instead found a significant effect of condition, responding in a similar pattern as left auditory cortex, also from 376–448 ms (sum t-value = 12.39; *p* = 0.033; Fig. 3C).

We additionally ran the same analysis in the inferior frontal gyrus (IFG), superior marginal gyrus (SMG) and middle temporal gyrus (MTG). Overall, we found no effects in IFG or SMG in either hemisphere (cf.^24^). Left MTG showed a main effect of condition in the left hemisphere from between 524 and 572 ms (sum t-value = 11.53; *p* = 0.034). There were no effects in the right middle temporal gyrus. Additional ROI analysis are presented in the supplementary materials.

Overall, the results of the ROI analysis suggest that adaptation to mispronounced speech is primarily supported by processes in frontal cortex. Sensory areas, by comparison, revealed a consistent increase in responses to substituted phonemes. Next we sought to test how information is passed from one region to another.

### Connectivity analysis

Previous theories suggest that listening to accented speech requires the recruitment of additional cognitive processes to facilitate comprehension^40^. Further, recent evidence suggests that not only activity in independent hubs, but also the efficient connection between them, is likely equally as important for successful processing. Based on this, we next sought to test whether the recruitment of additional cognitive processes, in response to non-canonical speech, may be reflected in additional or differential functional connections between brain areas.

**Figure 4:**
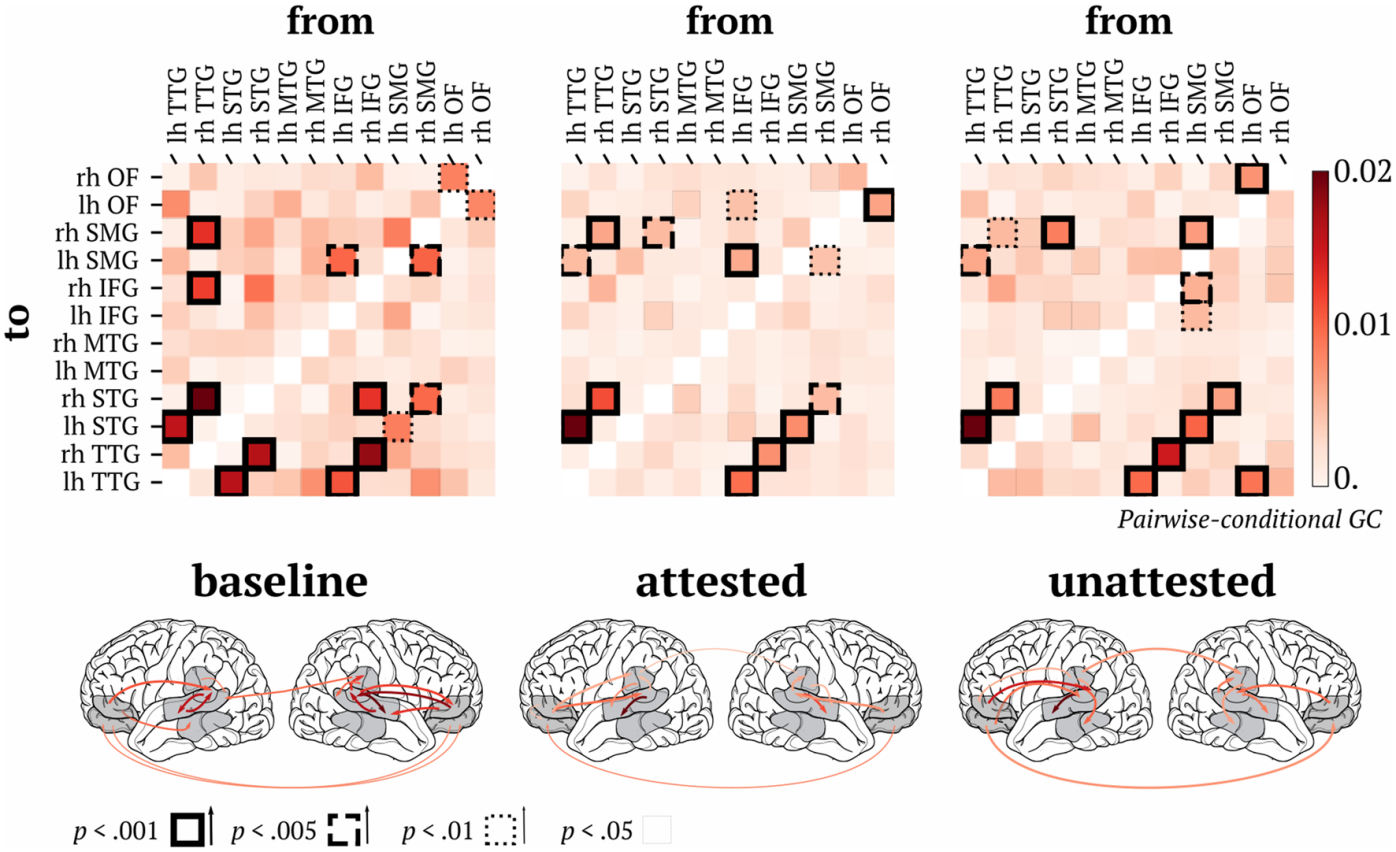
Connectivity patterns across regions bilaterally. In the matrices, the shade of red indicates the Granger causality value of the connections, and the border of the squares the significance level: solid bold line = *p* < .001; bold dashed line = *p* < .005; thin dashed line = *p* < .01; solid thin line = *p* < .05. The brain models underneath each matrix show the location of the connections with a significance of *p* < .01 or greater. The legend at the bottom indicates that the shade of the line corresponds to strength of the causal connection, using the same colorbar above, and the thickness to statistical significance.

To this aim, we ran a Granger causality analysis on our region of interest (ROI) data to determine the connectivity patterns between regions in our data. The complete set of connections is displayed in Fig. 4. Importantly, there are four patterns of results that we want to highlight. First, we found that the bilateral feedforward connection from primary auditory cortex (transverse temporal gyrus) to STG is significant (*p* < 0.001) for all three experimental conditions. This serves as a sanity check that our analysis is working as intended, given that it is known that these areas interact during normal auditory processing^50,51^. Second, we also find that there are also consistent connections across all conditions from auditory cortex to inferior frontal gyrus, bilaterally (*p* < 0.001) as well as from superior marginal gyrus to superior temporal gyrus, bilaterally (*p* < 0.001). Third, we only find a significant connection between STG and primary auditory cortex in the baseline condition (*p* < 0.001), and not in the other conditions. Finally, there is a significant connection between left orbito-frontal cortex and left primary auditory cortex exclusively for the unattested pronunciation condition.

## Discussion

The aim of the present study was to investigate the neural mechanisms supporting perceptual adaptation to systematic variations of speech. Here we test whether adaptation to non-canonical pronunciations occurs through the recalibration of early auditory processing (i.e. after adaptation, the acoustic signal gets directly classified as a different phonological category), or whether it is supported by a higher-order repair mechanism (i.e. the acoustic to phoneme mapping remains the same even after adaptation, hence the listener initially maps the sounds to the wrong category, and corrects this later). We operationalized this as testing whether the behavioral correlate of adaptation to non-canonical pronunciations manifests itself in the auditory cortices between 100 and 200 ms (supporting the recalibration hypothesis) or later activity (> 200 ms) in higher order areas (supporting the repair hypothesis).

Overall, our results support the repair hypothesis: the correlate of attunement emerges ~ 350 ms after phoneme perception in orbito-frontal cortex. Intuitively, one may have predicted that adaption would develop as a change from increased activation for non-canonical speech in the first exposures to baseline-like activity as a matter of exposure. However, what we found instead was that canonical speech decreased activity as participants were exposed to it while activity for non-canonical pronunciations (both attested and unattested) remained high throughout.

Further, we found that sensory regions are not modulated by exposure, and consistently elicit stronger responses to non-canonical as compared to canonical pronunciations (180–600 ms). Importantly, the connectivity *between* STG and primary auditory cortex bilaterally is significantly altered by non-canonical speech. This was true both for attested and unattested pronunciations, further highlighting the fact that we found no difference between these two conditions throughout. The combination of these results has critical implications for speech comprehension specifically, as well as auditory perception more generally.

### Role of frontal cortex

One of the critical findings of the present study is that activity for canonical speech decreases with exposure in the left frontal cortex, whereas responses to non-canonical speech remain relatively elevated throughout. Interestingly, this contrasted with results in the right prefrontal cortex, where we instead found a pattern of activity that mirrored left auditory cortex (i.e., activity for non-canonical speech was continuously higher than baseline responses).

Since regions of the prefrontal cortex have substantial anatomical connections to auditory areas^52,53^, there are anatomical grounds to consider them ideal candidates to modulate the operation of lower-level auditory areas. In fact, lateral portions of the prefrontal cortex have been previously reported to be involved in top-down resolution of ambiguity in the identity of speech sounds (e.g.,^54^), in speech segmentation^55^, and during the comprehension of connected speech (e.g.,^56–62^). Thus, it straightforwardly follows that the response we observe in the left orbitofrontal cortex is in fact reflecting top-down repair of the original (“incorrect”) phoneme categorization of the non-canonical sensory input into the “correct” phoneme categorization given the idiosyncrasies of the speaker.

The results on the right prefrontal cortex are qualitatively distinct though, as they merely show a reflection of the auditory cortex results. Previous research in non-human primates has shown that afferents from some early parts of the auditory system, presumed to carry information regarding auditory features of sounds, project this information to the prefrontal cortex^52,53,63,64^. Thus, it is likely that the result in the right orbitofrontal cortex is the manifestation of these projections.

In fact, differentiated neuronal responses have been previously found in the prefrontal cortex, some prefrontal neurons exhibiting responses to features in acoustic stimuli, while other neurons display task-related responses^63^. In our study, it would seem like the right prefrontal cortex is displaying the projection of basic auditory information, while the left is processing the task relevant information, the task being phoneme categorization for lexical identification in this case.

### Role of auditory cortex

The second critical result of this study is that non-canonical speech elicited consistently increased activity in auditory cortex. Unlike activity in frontal areas, these responses *did not wane* as a function of exposure. This result is consistent with a surprise signal in response to the mispronounced phoneme. Phoneme surprisal has been linked to modulation of activity around 200–300 ms in transverse temporal gyrus and superior temporal gyrus^29–31,65,66^. Relatedly, sensitivity to the phonotactics of the language has also been shown in similar cortical locations with a slightly longer latency^67,68^. The timing (200–600 ms) and location (STG) of our observed effects are consistent with these previous studies and posits surprisal signals, based on prior knowledge of phoneme sequence statistics, as a plausible source of heightened response to the mispronounced speech. Importantly, there were no effects of typicality at the auditory M100 component. This is likely due to the fact that this response reflects bottom-up processing, without considering the global lexical content of the word, and at the lowest-level, all the phonemes in our stimuli were well-formed—even the mispronounced ones—they were just placed in the “wrong” word.

Finally, we found that the within-hemisphere connection between STG and TTG, which was observable for canonical “baseline” speech, was not significant for either attested or unattested conditions. Future work should adjudicate between the following possible explanations: (1) the absence of robust lexical activation in the latter cases reduces the feedback connections from STG to primary auditory cortex; (2) reinforcement of “correct” acoustic–phonetic mapping is only present for correctly pronounced phonemes.

### Implications for models of effortful listening and accented speech

The general model of effortful listening and accented speech proposed by 40 suggests that the comprehension of accented speech is cognitively effortful because there is a mismatch between the expectations of the listener and the received signals. Further, they intuitively suggest that getting used to a given accent should automatically change the expectations of the listeners, hence reducing the mismatch between expectations and input. This should in turn lower the demand for compensatory signals and associated cognitive effort.

Our experiment did not straightforwardly match these predictions: the signals to both kinds of non-canonical speech in auditory cortex remained constant throughout. This opens two possibilities. The first is that this effortful listening model is inaccurate in assuming an effect of social expectation in auditory perception. At the lowest level, phonemes will inevitably be categorized based on the physical properties of the signal, and not based on the intended phoneme of the speaker. Thus, there is no escaping automatic predictions, and subsequent prediction error signals, in auditory cortex.

The second possibility is that there is a dissociation between the timelines of behavioral and neural measures of adaptation. That is, even if behaviorally adaptation occurs quickly, the underlying neural processes will adapt at a slower pace and will necessitate two stages. Although our study did not contain enough exposure to dissociate between these two stages, theoretically, this is how such a mechanism would work. First, after a short exposure to non-canonical pronunciations (for instance an accent, although it does not have to be), listeners will be able to fully understand, as shown by behavioral accuracy measures. However, achieving this intelligibility will require additional cognitive effort, as reflected in (1) elevated difficulty in understanding^69,70^ and (2) slower processing for accented speech^71,72^, and it will rely on high prediction error *correction* signals. Further down the line, the effort required to comprehend will reduce: we predict that at this stage there will be a decrease in the error prediction signal, and a reestablishment in the functional connections between STG and primary auditory cortex, leading to a pattern of activity more similar to that of the perception of canonical speech. Unfortunately, our results do not adjudicate between these two possibilities because our experiment gave participants relatively little exposure to non-canonical speech.

## Conclusion

In all, this paper constitutes the first characterization of the neural markers of auditory adaptation to systematic phonological variations in speech during online speech perception. These findings contribute a critical piece to our hitherto poor understanding of how the brain solves this puzzle, which was reduced to behavioral descriptions of this phenomenon, and anatomical structural differences for better perceivers^73^. We unveil that understanding mispronounced speech relies on prefrontal signals and propose that this only represents the first stage on the road to attunement, which by hypothesis subsequently relies on lower level adaptation to phonological classification in sensory areas.

## Supplementary Information

Supplementary Information
