# Supplementary material for: Adaptation to mis-pronounced speech: evidence for a prefrontal-cortex repair mechanism
Source: Sci Rep. 2021 Jan 8;11:97. doi: 10.1038/s41598-020-79640-0 (PMC7794353; doi:10.1038/s41598-020-79640-0)

# Perceptual adaptation to non-canonical pronunciation: prefrontal cortex aids attunement in auditory cortices

Esti Blanco-Elorrieta[1,3*], Laura Gwilliams[1,3*], Alec Marantz[1,2,3], & Liina Pylkkänen[1,2,3]
* denotes equal contribution

1. Department of Psychology, New York University, New York, NY 10003, USA
2. Department of Linguistics, New York University, New York, NY 10003, USA
3. NYUAD Institute, New York University Abu Dhabi, Abu Dhabi, P.O. Box 129188, UAE

# Supplementary ROI analyses

Figures below show the average response time course in different cortical regions that have been implicated in accented speech processing in previous studies. Data for each condition are split into the first half of exposures (0 in the legend) and second half of exposures (1 in the legend). The figure setup shown here is the same as shown in Figure 3 of the main manuscript.

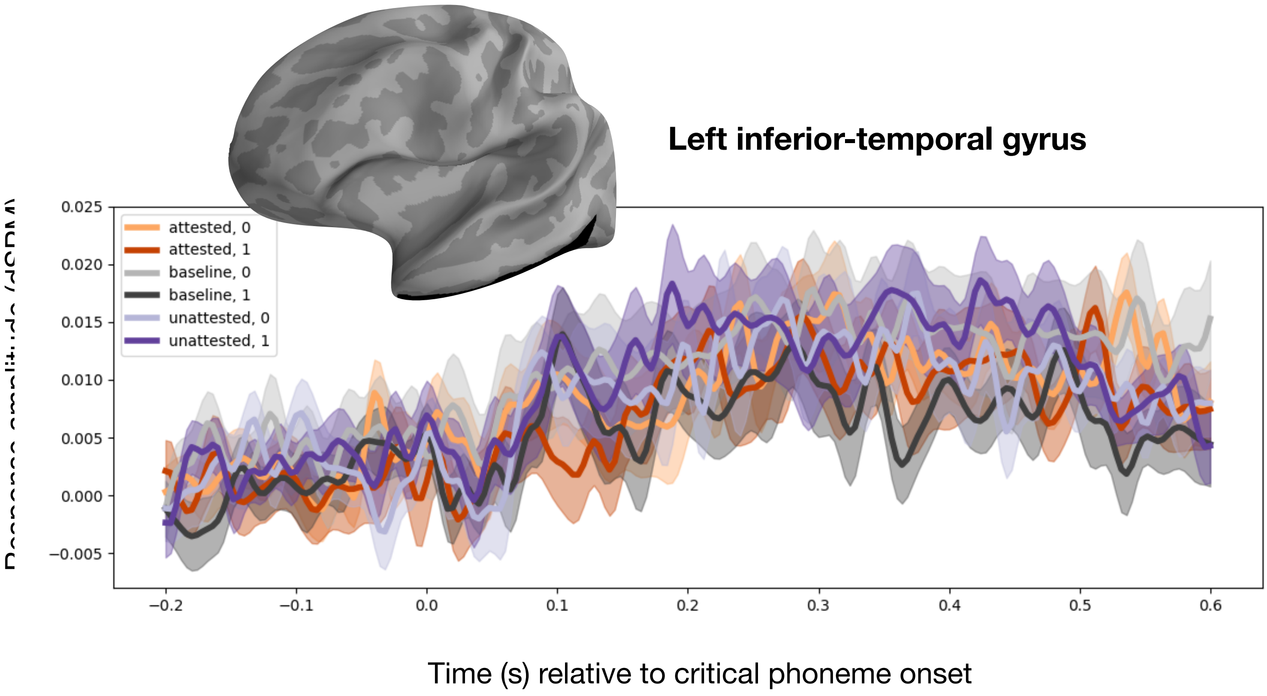


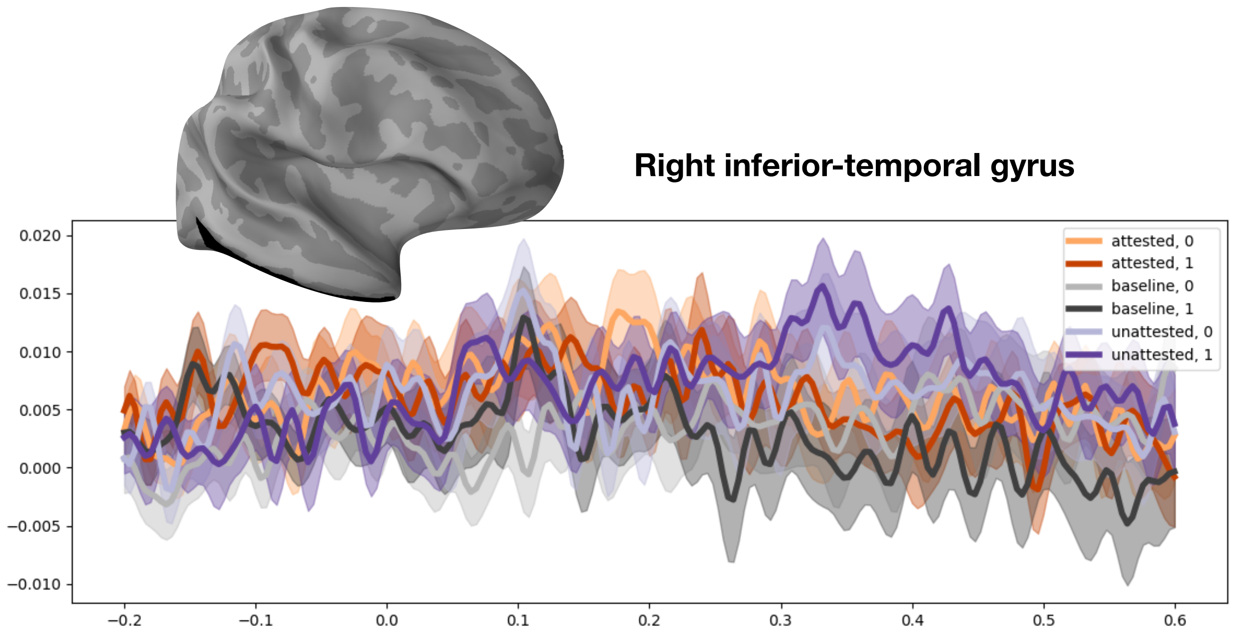

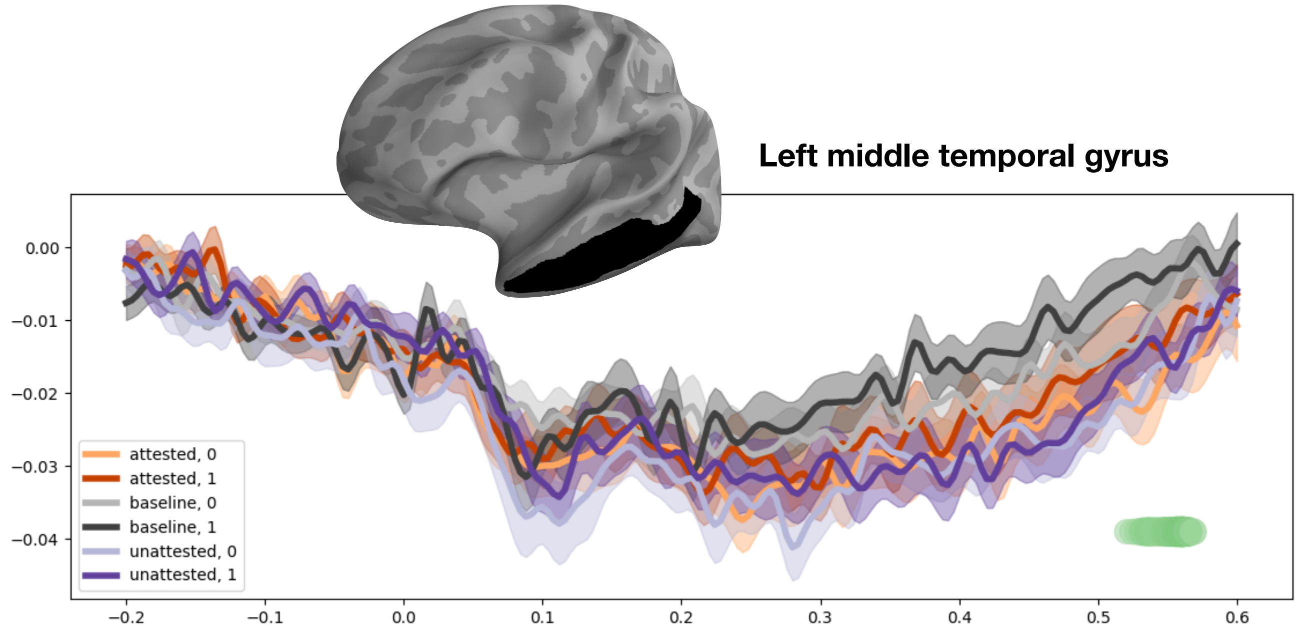
Left middle temporal gyrus
0.00
−0.01
−0.02
−0.03
−0.04
attested, 0
attested, 1
baseline, 0
baseline, 1
unattested, 0
unattested, 1
−0.2
−0.1
0.0
0.1
0.2
0.3
0.4
0.5
0.6

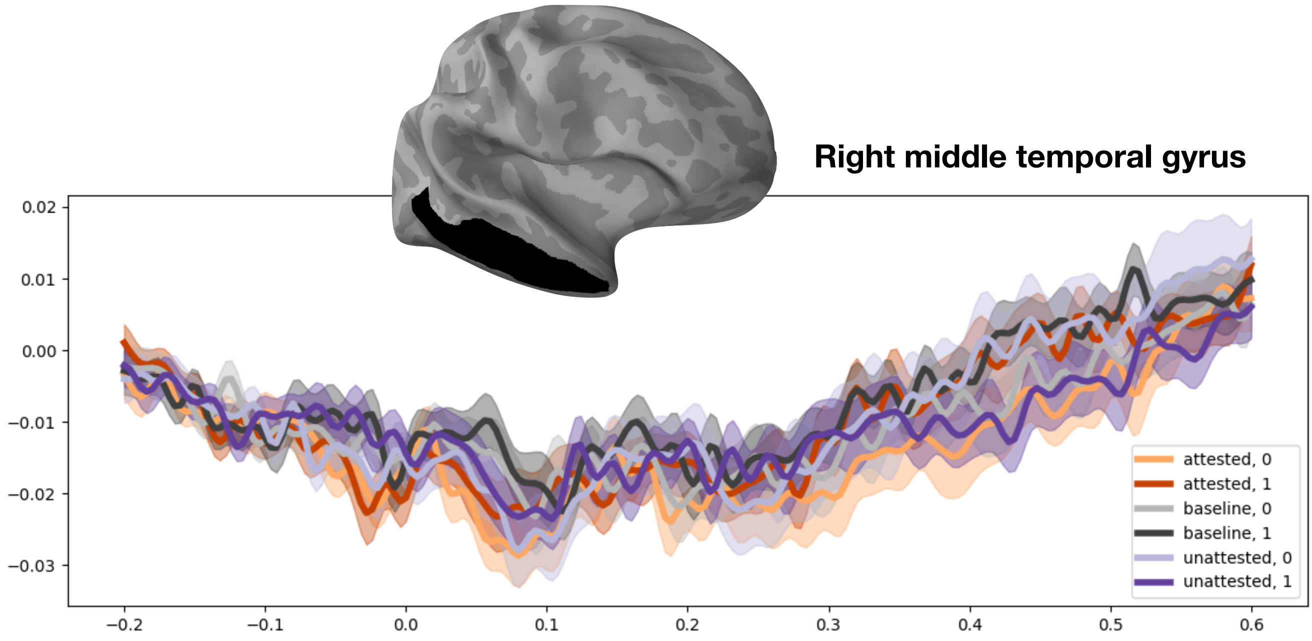
Right middle temporal gyrus
0.02
0.01
0.00
−0.01
−0.02
−0.03
attested, 0
attested, 1
baseline, 0
baseline, 1
unattested, 0
unattested, 1
−0.2
−0.1
0.0
0.1
0.2
0.3
0.4
0.5
0.6

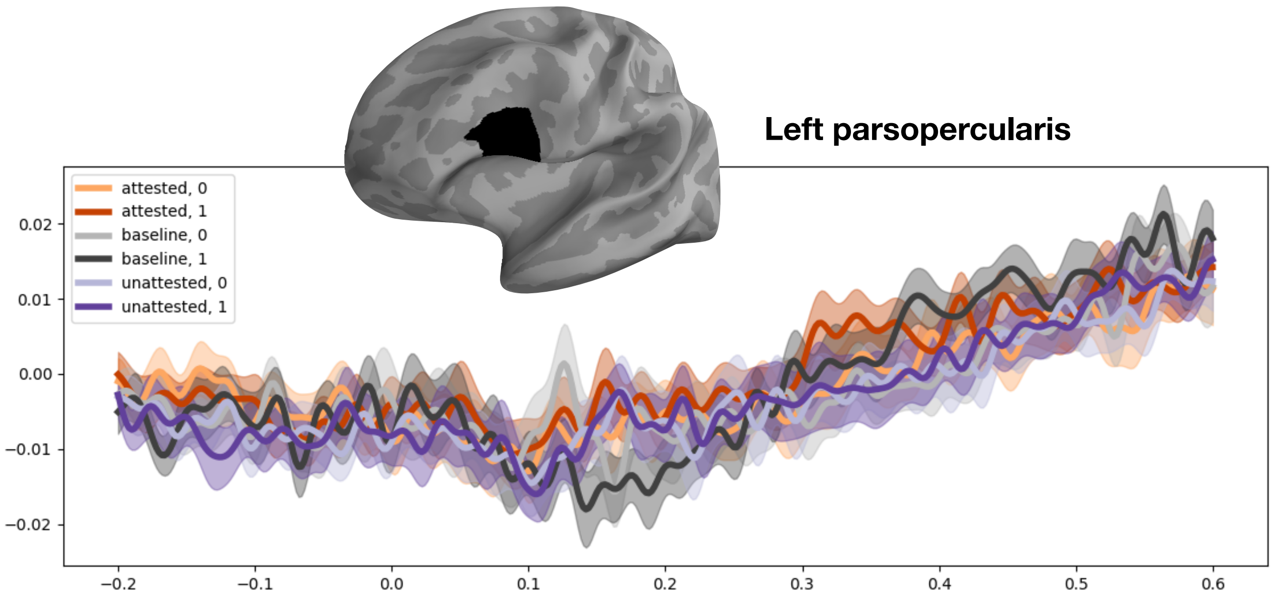
Left parsopercularis
attested, 0
attested, 1
baseline, 0
baseline, 1
unattested, 0
unattested, 1
0.02
0.01
0.00
−0.01
−0.02
−0.2
−0.1
0.0
0.1
0.2
0.3
0.4
0.5
0.6

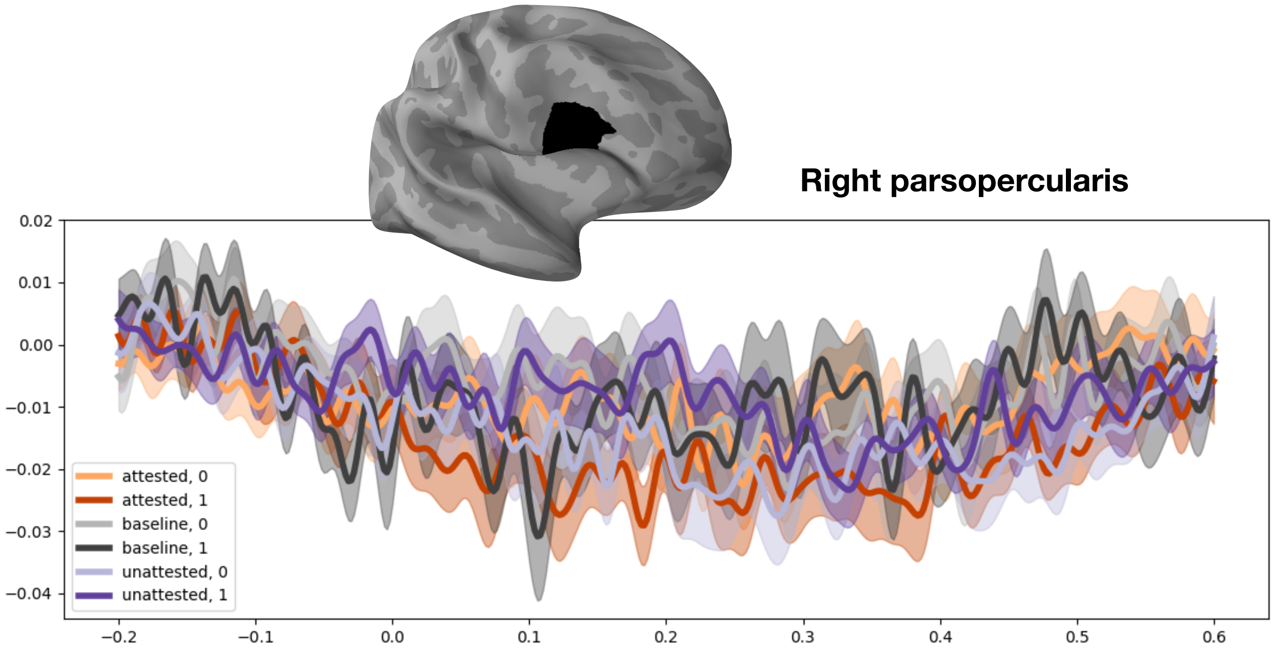
Right parsopercularis
0.02
0.01
0.00
−0.01
−0.02
−0.03
−0.04
attested, 0
attested, 1
baseline, 0
baseline, 1
unattested, 0
unattested, 1
−0.2
−0.1
0.0
0.1
0.2
0.3
0.4
0.5
0.6

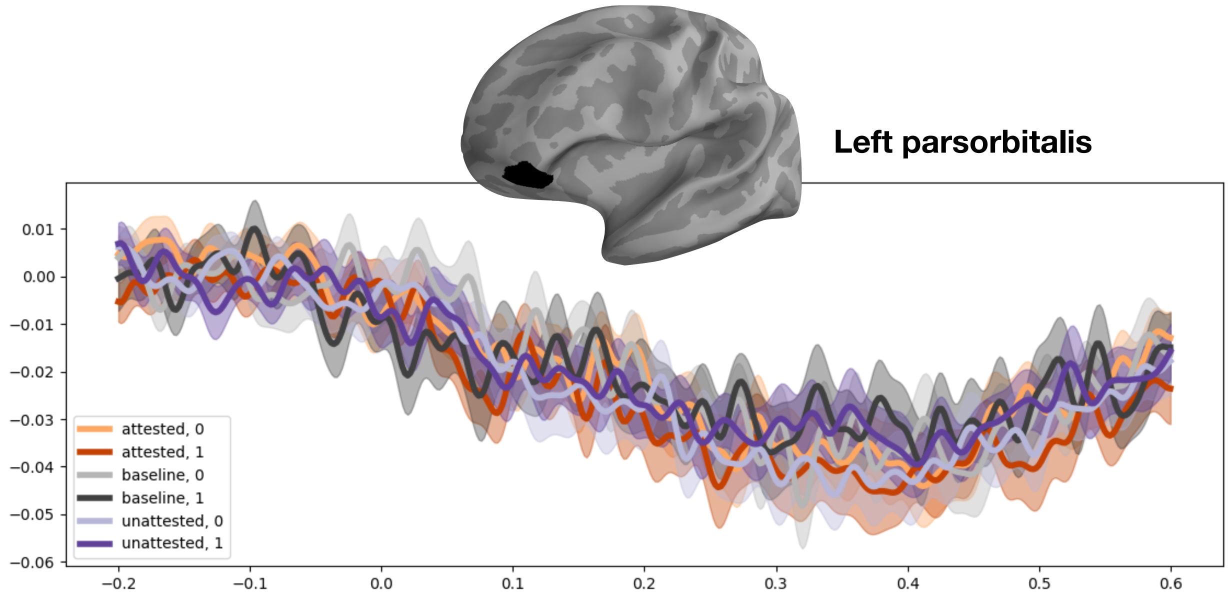
Left parsorbitalis
0.01
0.00
−0.01
−0.02
−0.03
−0.04
−0.05
−0.06
−0.2
−0.1
0.0
0.1
0.2
0.3
0.4
0.5
0.6
attested, 0
attested, 1
baseline, 0
baseline, 1
unattested, 0
unattested, 1

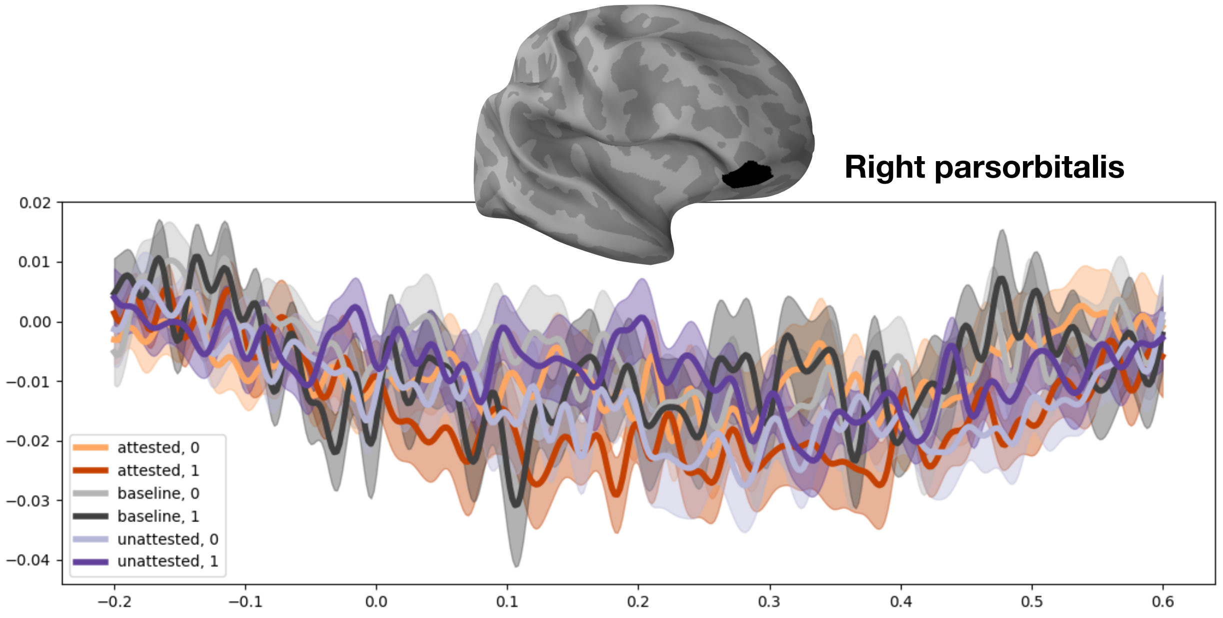
Right parsorbitalis
0.02
0.01
0.00
−0.01
−0.02
−0.03
−0.04
−0.2
−0.1
0.0
0.1
0.2
0.3
0.4
0.5
0.6
attested, 0
attested, 1
baseline, 0
baseline, 1
unattested, 0
unattested, 1

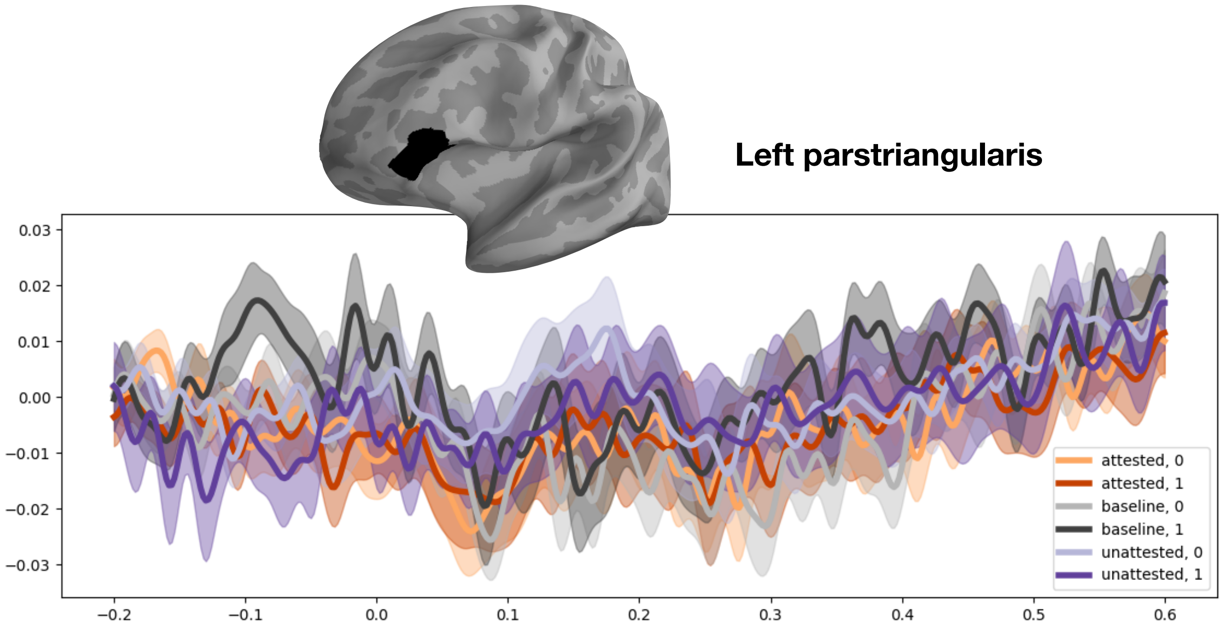
Left parstriangularis
0.03
0.02
0.01
0.00
−0.01
−0.02
−0.03
−0.2
−0.1
0.0
0.1
0.2
0.3
0.4
0.5
0.6
attested, 0
attested, 1
baseline, 0
baseline, 1
unattested, 0
unattested, 1

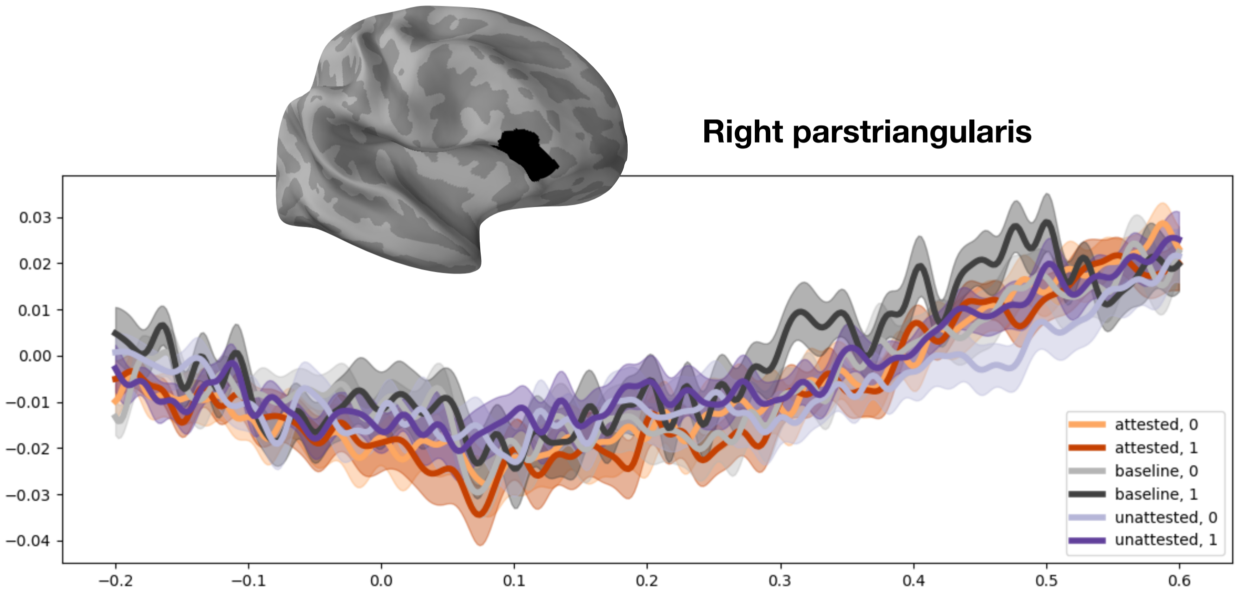
Right parstriangularis
0.03
0.02
0.01
0.00
−0.01
−0.02
−0.03
−0.04
−0.2
−0.1
0.0
0.1
0.2
0.3
0.4
0.5
0.6
attested, 0
attested, 1
baseline, 0
baseline, 1
unattested, 0
unattested, 1

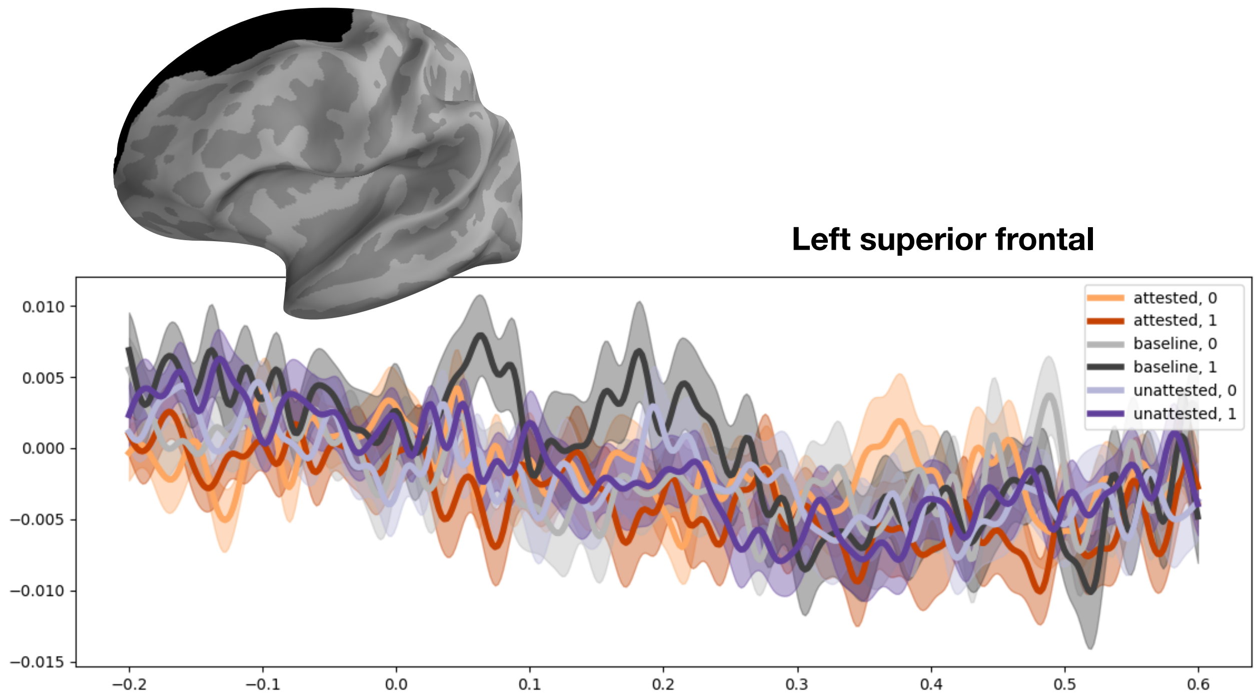
Left superior frontal
attested, 0
attested, 1
baseline, 0
baseline, 1
unattested, 0
unattested, 1
0.010
0.005
0.000
−0.005
−0.010
−0.015
−0.2
−0.1
0.0
0.1
0.2
0.3
0.4
0.5
0.6

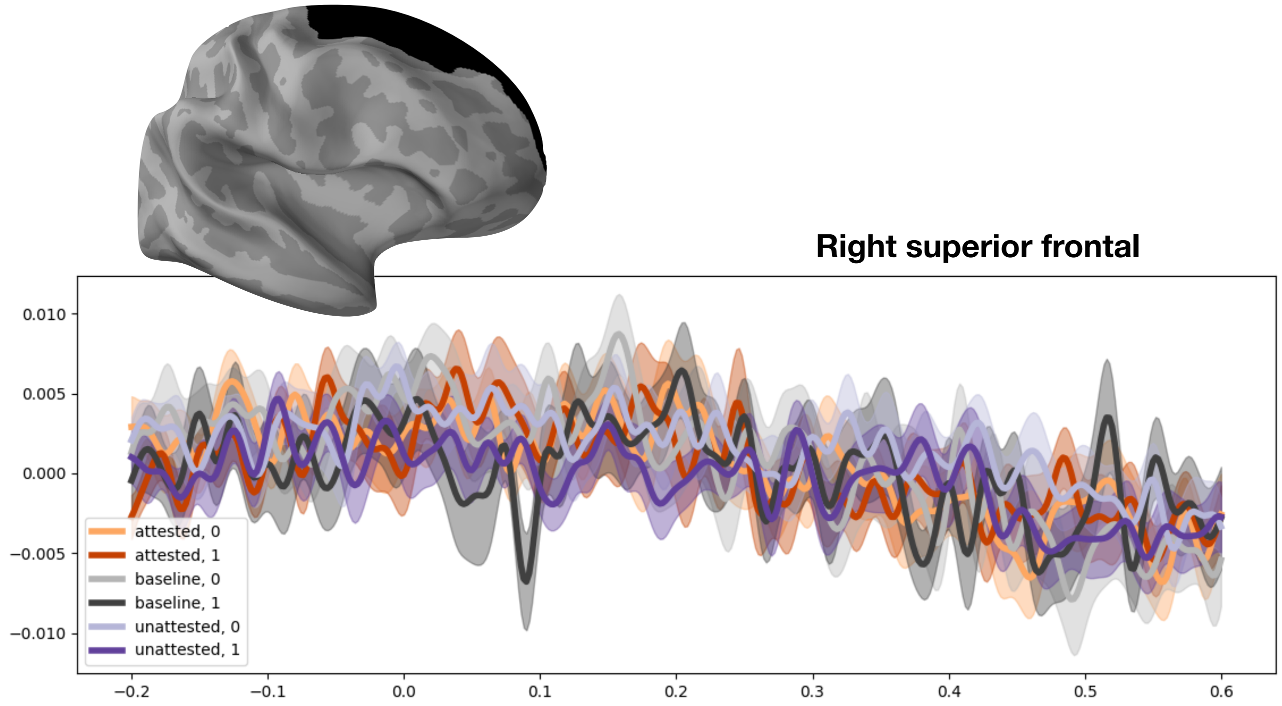
Right superior frontal
attested, 0
attested, 1
baseline, 0
baseline, 1
unattested, 0
unattested, 1
0.010
0.005
0.000
−0.005
−0.010
−0.2
−0.1
0.0
0.1
0.2
0.3
0.4
0.5
0.6

## Left supramarginal gyrus

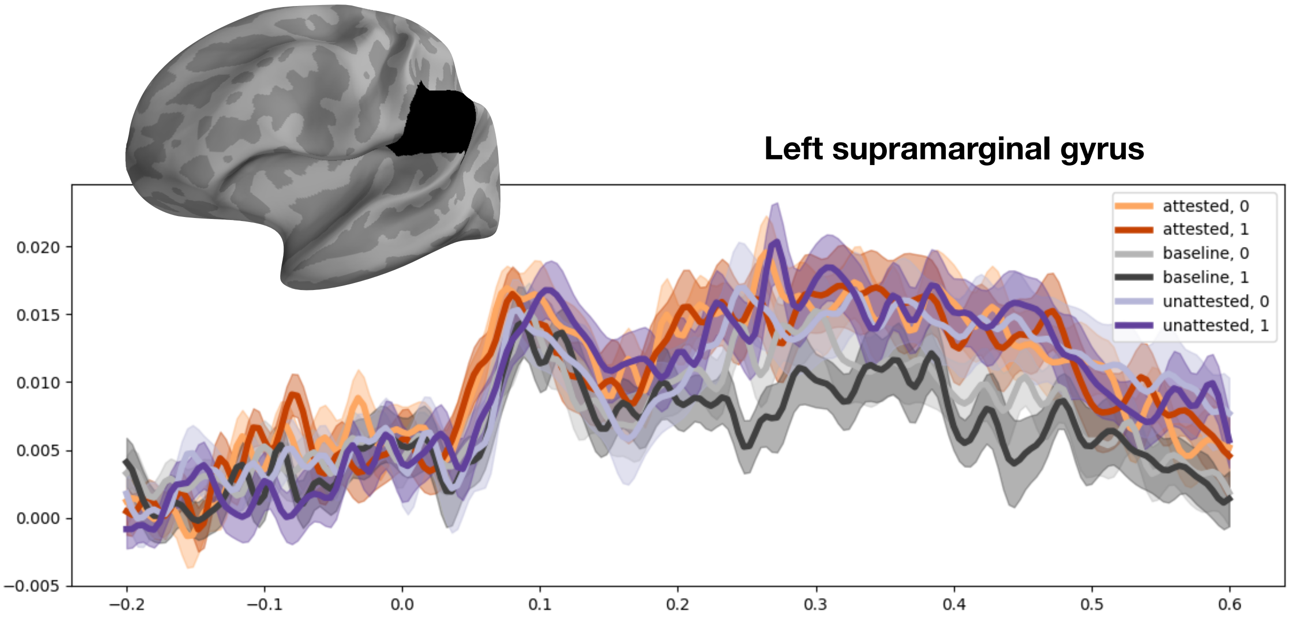


## Right supramarginal gyrus

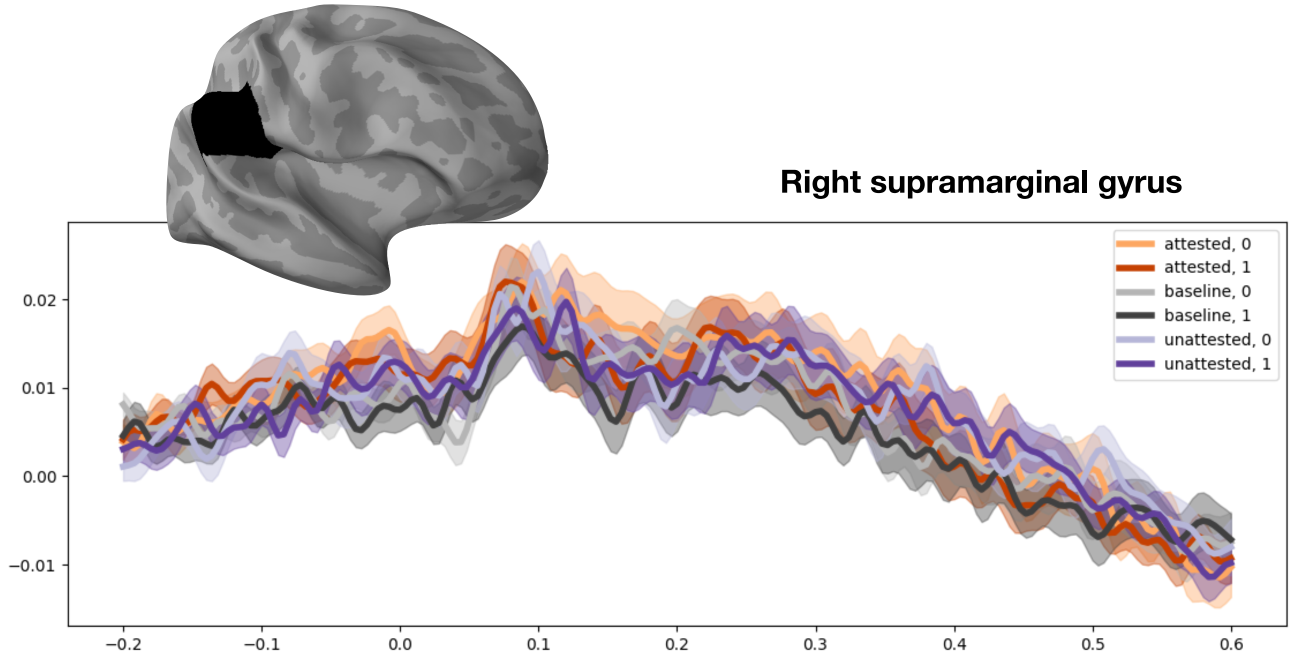

Supplement: Supplementary file 1 — Supplementary Information [file 41598_2020_79640_MOESM1_ESM.pdf]
